# Benefits of Training Volunteers in Mental Health Promotion Within Organisations Employing People with Disabilities: Preliminary Results

**DOI:** 10.3390/healthcare14193329

**Published:** 2026-10-06

**Authors:** Santiago Gascón-Santos, Agustín Albesa, Ricardo Fueyo-Díaz, Ana Isabel Marqués Fernández, Yago Pérez-Montesinos

**Affiliations:** 1 Department of Psychology and Sociology, University of Zaragoza, C/Atarazanas, 2, 44003 Teruel, Spain; albesa@unizar.es (A.A.); rfueyo@unizar.es (R.F.-D.); amarques@unizar.es (A.I.M.F.); yagoperezmontesinos@gmail.com (Y.P.-M.); 2 Faculty of Social Sciences and Humanities, University of Zaragoza, C/Atarazanas, 2, 44003 Teruel, Spain

**Keywords:** functional diversity, mental health training, health promotion, prevention, suicide

## Abstract

**Highlights:**

**What are the main findings?**

- Staff training on mental health promotion (particularly in organisations with employees with disabilities) can have a positive impact across the whole organisation.
- These improvements in mental health indicators may begin to be observed six and twelve months after the programme is launched, but it is assumed that staff will require ongoing training and monitoring over time.

**What are the implications of the main findings?**

- Suicide prevention can, to a large extent, be based on an effective policy for promoting mental health, both in companies and in any other type of organisation.
- Mental health is the responsibility of the community, but also of each individual.

**Abstract:**

**Background:** Psychosocial factors present in various work environments can pose a risk to workers’ health, but those with some form of disability may be more vulnerable and more severely affected by these issues. Objective: The goal of this study is to assess whether training employees to act as health promoters, as well as providing them with follow-up support, can help to improve health outcomes among other workers and serve as a link between them and healthcare professionals. **Method**: Initially, 148 employees were recruited from five centres belonging to the same company, in which the majority of staff had some form of disability or were at risk of social exclusion. Three centres were randomly assigned to the intervention group and two centres formed the control group (waiting list). Measurements were taken at three time points (baseline, at six months and at twelve months). Initially, 82 people participated from the intervention group, and at the two subsequent time points, 69 and 55 respectively. From the control group, 49 employees participated in the baseline assessment and, at the two subsequent time points, 37 and 32 respectively. The following instruments were used: (1) a demographic and employment data form; (2) the General Health Questionnaire (GHQ-12); (3) the Maslach Burnout Inventory (MBI General Questionnaire); and (4) five items from the Columbia Scale for the Assessment of Suicidal Severity. **Results:** The intervention group showed improvements in all variables over time and also in comparison with the control group. These improvements were statistically significant in terms of general health and in the dimensions of cynicism and professional efficacy. **Conclusions**: The establishment of mental health promotion teams within organisations can help to improve key aspects of employees’ psychological wellbeing, which encourages further research in this field to investigate the effectiveness of structured and sustainable interventions.

## 1. Introduction

Social enterprises and other supported-employment organisations have been established in several countries to facilitate access to work for people with disabilities. Psychosocial working conditions are associated with burnout and other adverse outcomes [1]. Workers with disabilities may additionally experience barriers, discrimination and variation in managerial support; however, the available literature does not justify treating this population as homogeneous [2].

Mental disorders account for a substantial share of global disease burden [3]. Suicide caused more than 900,000 deaths worldwide in 2024 [4], and prevention requires attention to both risk and protective factors [5]. At the same time, mental-health services in many countries face large treatment gaps, uneven coverage and limited resources [6].

Mental health depends on interactions between individual characteristics and social conditions. Promotion therefore includes helping people adopt healthy ways of life and creating environments conducive to health [7]. Public education and early intervention form part of this broader approach [8]. A number of studies have been carried out which show how appropriate training in psychological first aid for non-specialist staff can provide humane, practical and immediate support following traumatic events, although it cannot be taken for granted that data from emergency settings are sufficient to demonstrate its effectiveness in ordinary workplaces [9].

Workplaces are important settings for mental-health promotion because they provide sustained access to working populations and allow organisational as well as individual interventions [10]. Psychosocial work factors are associated with stress, exhaustion and safety outcomes, particularly in high-risk industries [11]. The third European Survey of Enterprises on New and Emerging Risks documents the widespread organisational relevance of psychosocial risks, including difficult interactions and time pressure; it is an enterprise survey and should not be interpreted as a diagnostic estimate of worker stress [12].

Our research group is keenly aware of the issue of suicide, particularly following a call from the regional government in 2022–2023 to develop initiatives that might help alleviate this scourge. Following a period of discussion between policy-makers and professionals in the fields of medicine, psychology and psychiatry, it was concluded that, in addition to other interventions in crisis situations, the issue should be tackled through a policy of continuous mental health promotion across all sectors (educational institutions, the workplace, society, the media, etc.), with a focus on providing training to prevent problems such as bullying at school, work-related stress, loneliness amongst older people and misinformation about mental health. Although it may be difficult to quantify the contribution made in each of these areas, we are pressing ahead with the project in the hope of reducing the number of suicides in all of them in the coming years.

The present article forms part of a broader workplace mental-health and suicide-prevention project. The programme sought to involve senior managers, middle managers and employees in recognising signs of distress, offering initial support, facilitating access to professional services and promoting well-being. Current guidance recommends organisational prevention, manager training and support for workers experiencing mental-health difficulties [13]. Within that framework, mental health promoters were conceived as an accessible internal resource, not as substitutes for healthcare professionals.

The study forms part of a wider research project, running for six years (2024–2029), aimed at preventing suicide through mental health promotion initiatives. These initiatives are based on the ongoing training of lay helpers in workplaces, educational settings, senior citizens’ associations, etc., who act as observers and facilitators within their communities, providing mental health first aid (primarily by offering support, promoting healthy activities and referring people to health services within the organisation or the local community). Specifically, in workplace settings, baseline measurements are taken at the start of the first six months and annually, covering variables relating to general health, dimensions of burnout, and suicidal ideation. Recognising that changes in mental health data occur gradually, the design is based on monitoring these variables at different points over time.

The aim was for a small number of employees to volunteer for the training and subsequent follow-up. The ratio in other institutions assessed was one staff member for every 10–15 employees. The distinctive feature of these inclusive organisations was that human resources managers put themselves forward (as revealed in qualitative interviews, they were the most concerned about psychosocial issues amongst the workforce, such as demotivation, lack of engagement or absenteeism). As in the other organisations in the study, the support officers received training in mental health first aid (detecting possible emotional and behavioural changes, establishing and strengthening personal channels of communication, knowing how to provide social support and, in particular, being familiar with the various health services and knowing how to refer people to them). It is often said that training in the use of defibrillators or in how to respond to potential accidents can save lives whilst waiting for the emergency services to arrive. The aim was not to turn these officers into experts, but to ensure they were familiar with first aid and knew how to refer cases to the health services. The aim of the study was to identify preliminary findings that might support the use of training in conjunction with other measures aimed at improving mental health.

In other words, the aim was to describe the changes that took place during the first twelve months following the programme’s launch and to compare the organisations that benefited from the promotion programme with those that received standard information activities. Given that only five organisations were selected (specifically within the sector of integration enterprises), the analyses and hypotheses have been regarded as exploratory in nature.

- Exploratory hypothesis 1: Employees in organisations implementing the promoter programme would show favourable changes over time in psychological distress, burnout dimensions and suicidal ideation.
- Exploratory hypothesis 2: Changes would be more favourable in the intervention organisations than in the comparison organisations.

## 2. Materials and Methods

This article presents the results obtained at a company whose workforce consists predominantly of workers with disabilities and/or at risk of social exclusion. This is a longitudinal study involving two groups, with baseline measurements and follow-up assessments at 6 and 12 months. Data collection took place between October 2024 and December 2025.

### 2.1. Participants

Work was carried out in a company with several sites of social integration, partly funded by the government. The workforce consisted of people with some form of disability or at risk of social exclusion. These are not identical concepts, but they share characteristics in terms of employment-related factors. Workers indicated whether they had a disability and, in some cases, specified what type it was. For example: visual impairment (congenital cataracts), or intellectual disability (Down’s syndrome). In cases of those at risk of social exclusion, the majority were migrants who were learning the language (particularly the written form) and who required assistance from the researchers to complete the questionnaires, or who had been attending a rehabilitation programme. None of the participants were pressured to provide more information than they wished to give. Therefore, although we were able to ascertain the details of each case, no further information beyond that provided in the questionnaires was included in the text. Workers may remain in this type of integration institution for a maximum of four years. During this period, if a position is found for them in other companies, they are offered that opportunity.

**Figure 1:**
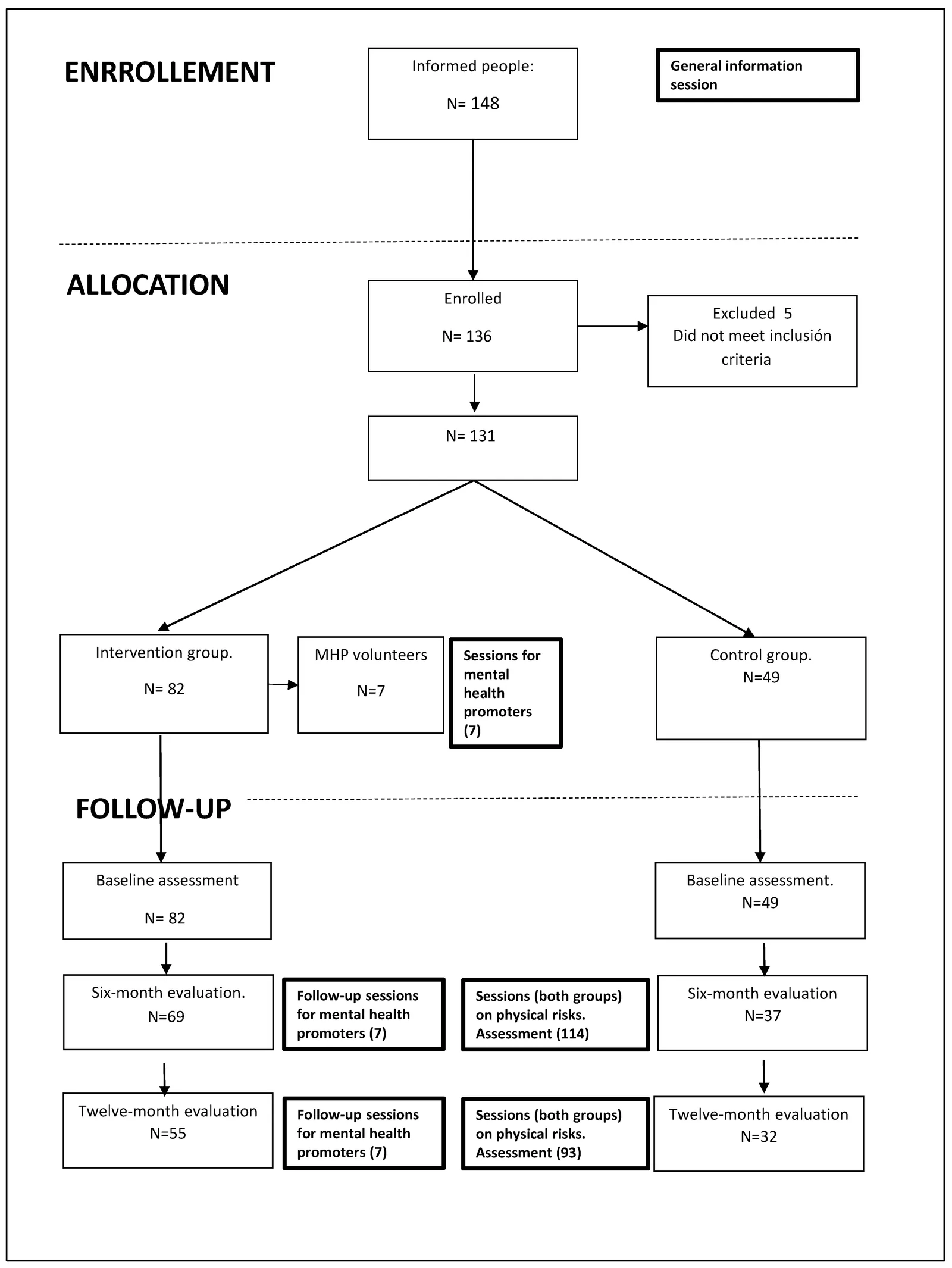
Flow chart of participants during the study.

The initial information sessions were attended by 148 people, including staff and managers from the participating organisations. The organisations were randomly assigned (three to the intervention group and two to the control group). Of the 136 participants who completed the pre-assessment questionnaires, five were excluded for failing to meet the criteria (four due to missing identification codes and one due to a diagnosis of a mental disorder). In the first phase, responses were obtained from 82 employees from the organisations in the intervention group and 49 employees from the organisations in the control group. From the intervention group, 69 responded to the six-month assessment and 55 to the 12-month assessment. From the control group, 37 employees and managers completed the questionnaires at six months and 32 at 12 months. All participants were assigned a personalised and anonymous code, and only the responses of those who took part in all three measurement points were taken into account (Figure 1).

The inclusion criteria for participation in either group were: (a) being aged between 18 and 60, (b) having been employed by the company for at least one year. The exclusion criterion was a diagnosis of a mental health disorder.

Regarding mental health promotion agents (MHPAs), all employees were offered the opportunity to volunteer to receive specific training and act as MHPAs, regardless of their position within the organisation. Six of the seven people who volunteered were human resources managers. This surprised the researchers, as studies carried out in other workplaces had shown that volunteers came from all levels of the workforce. In response to our queries, we were told: (1) most employees (particularly foreign nationals) felt somewhat self-conscious about working with researchers; (2) human resources staff were particularly keen to collaborate on the study because they believed there was low employee engagement and that employees might be at greater risk of psychosocial harm. All of this was discussed with managers and some staff members. Ultimately, the results did not confirm worse scores for burnout or general health compared with other organisations studied by our group.

### 2.2. Instruments

#### Measures

At the initial assessment and during subsequent follow-ups, participants completed the following questionnaires.

- Demographic and employment form: gender, age, disability, living arrangements and length of service.
- General Health Questionnaire GHQ-12. The GHQ-12 screens recent psychological distress and does not diagnose a specific mental disorder [14]. The manuscript reports Likert scoring from 0 to 3. The exact coding rule and any threshold used must be reported in the final statistical revision. Internal consistency in this study was α = 0.77.
- Maslach Burnout Inventory-General Survey. The 16-item MBI-GS assesses exhaustion, cynicism and professional efficacy using frequency responses from 0 to 6 [15,16]. Higher exhaustion and cynicism and lower professional efficacy indicate a less favourable burnout profile. Internal consistency was α = 0.88, 0.70 and 0.76, respectively.
- Columbia-Suicide Severity Rating Scale (C-SSRS; Posner et al., 2011) [17]. These questions assessed the severity of possible suicidal ideation on a 5-point ordinal scale (ranging from 0 = absence of thoughts or desire to die to 5 = suicidal ideation with a concrete plan and intention).

### 2.3. Training

The training consisted of workshops delivered by professionals in the fields of psychology, psychiatry and occupational health, comprising 12 sessions of 90 min each (theoretical presentation and discussion), with a total duration of 18 h and a theoretical–practical approach, through which aspects relating to mental health in everyday life were defined and explored.

The training focused on recognising other people’s emotions and behaviours. It centred on understanding that there are certain indicators which may point to mental health issues (withdrawal, isolation, changes in eating or sleeping habits, etc.). We worked on ways to offer and provide social support whilst respecting the other person’s boundaries: across various sessions, we took a cross-cutting approach to developing communication techniques (training in empathy, intuition, respectful treatment and assertiveness skills). Emphasis was placed on when and how to contact the research team in case of doubt and, above all, on familiarising participants with mental health organisations and institutions (ranging from the company’s own healthcare staff to community services and support groups for different groups of people affected by mental health issues within the city). Examples of crisis support were discussed: what to do, who to turn to, and so on. In short, this is mental health first aid training, in the same way that people are taught how to use a defibrillator or how to respond to an accident or a small fire.

In the follow-up sessions, these explanations were expanded upon, drawing on real-life situations in which such incidents had occurred.

The intervention programme is briefly described in Table 1.

During these sessions, we worked with a team of volunteer officers to identify risk factors and protective factors, as well as how these issues influence mental health, both directly and indirectly. Emphasis was placed on understanding emotions and their manifestations, on cognitive functioning, and on promoting healthy lifestyles. Particular attention was paid to the resources offered by the healthcare system and the occupational health service, as well as by associations and other organisations. In short, the aim was to provide the officers with basic training in psychological support.

We must emphasise that this was not a particularly unwell population. Rather, like any population, it was at risk of experiencing symptoms and signs of declining mental health. As noted in the Section 5, health-related variables take time to manifest themselves and also to disappear. The aim of this preliminary study was not to intervene with people with mental health disorders, but rather to carry out ongoing mental health promotion to make it more difficult for such disorders to develop.

### 2.4. Procedure

The five centres belong to a company. It was found that the two groups did not differ significantly in terms of socio-demographic variables at the start of the study. The aim was to observe possible changes in health variables within those centres where staff who had received training in this area were working. As this was a long-term study conducted across various institutions (educational, workplace and social), we regard these initial findings as preliminary results of the study.

Both in the general study and in the specific case presented here, the aim was to observe how the results evolved during the first year of the study. It was considered that appropriate training delivered by mental health professionals, as well as participation in the network and follow-up by the research team, would contribute to improving health indicators and reducing potential suicidal ideation; this is expected to be further confirmed over the next five years.

In both groups, all staff were invited to attend, on a voluntary basis, an information session on mental health, psychosocial factors at work and the risk of suicide, and were given the opportunity to discuss these issues with the other attendees. Once the centres in the intervention group had been selected, a team of seven volunteer mental health promoters was formed from among their staff. These promoters received training in mental health and took part in subsequent exchange and follow-up activities. During these sessions, they were provided with information about the Mental Health Programme (MHP), what it involved and who could participate as promoters. Those who volunteered were asked to commit to attending all 12 sessions. Subsequently, the MHP agents took part in follow-up sessions every two months and were given the opportunity to contact the research team to clarify any queries or receive support in crisis situations.

The initial session on mental health was the same across all five centres. During two follow-up sessions, brief talks were given on the prevention of physical risks and on recommendations for good posture at work, after which the questionnaires were administered. It is possible that the responses reflect not only the efforts of the health promoters but may also be influenced by the level of support perceived from management.

Once the study was complete, the centres in the control group were invited to receive the same training as the intervention group regarding MHP Agents.

### 2.5. Statistical Analysis

The Kolmogorov–Smirnov normality test was applied to assess the distribution of the variables. As these did not follow a normal distribution, non-parametric tests were used. As there were dropouts at the various time points and the participants responded using a code, a list-based exclusion was carried out, completely excluding subjects who did not complete all three time points. Hedges’ g coefficient was calculated as a measure of effect size. The significance level was set at *p* < 0.05, and all statistical analyses were performed using SPSS software IBM Corp. (2013, Armonk, NY, USA).

## 3. Results

Table 2 describes the characteristics of the total sample and compares the intervention and control groups. Although the cohort initially comprised 131 participants, only the scores obtained by the 87 subjects who responded at all three measurement time points were analysed (55 participants in the intervention group and 32 in the control group).

In the intervention group, 52.7 percent were women, whilst in the control group the figure was 56.2 percent. The mean age was 40.31 (SD 4.55) in the intervention group, whilst in the control group it was 41.97 (SD 7.22). The majority of employees had some form of disability, the most common being physical and sensory disabilities, as shown in the table. No statistically significant differences were observed between the two groups in the main variables of the study.

Table 3 provides information on the changes observed in the groups at the three measurement points. Although improvements were observed over time within the intervention group in terms of general health, emotional exhaustion, cynicism, professional efficacy and suicidal ideation, these were not statistically significant in all cases. Statistical significance was found in the cynicism dimension, X2(2, N = 55) = 6.83, *p* = 0.050, and in the professional efficacy dimension, X2(2, N = 55) = 5.72, *p* = 0.033. Within the control group, no statistically significant changes were observed in any of the variables studied.

With regard to the comparison of measures between the two groups, the differences found were also in line with expectations, but these differences were only statistically significant in terms of general health (T2–T1; *p* = 0.001); in the cynicism dimension (T2–T1; *p* = 0.05) and (T2–T0; *p* = 0.047); and in the professional efficacy dimension (T2–T0; *p* = 0.036).

## 4. Discussion

The results appear to show some positive changes over time in the intervention group. However, these changes should be interpreted with caution and understood as preliminary findings or as potentially favourable trends. Hypothesis 1 was partially confirmed. Employees at centres where mental health promoters were active showed improvements across the three measurement points in the variables studied, although these were only statistically significant for the variables of cynicism and professional efficacy. Regarding hypothesis 2, an improvement was also observed in the intervention group compared with the control group across all variables, and these differences were statistically significant in terms of general health and the burnout dimensions of cynicism and professional efficacy.

As we have indicated, this was not a particularly unwell population. Rather, like any population, it was at risk of experiencing symptoms and signs of deteriorating mental health. As we also noted in the text, health-related variables take time to manifest themselves and also to disappear. The aim of this preliminary study is not to intervene with people with mental health disorders, but rather to promote mental health so that such disorders are less likely to develop.

The descriptive pattern was favourable for some outcomes, and the initial analysis identified specific differences in general health, cynicism and professional efficacy. However, the improvements were not statistically significant across all aspects, and neither burnout nor suicidal ideation showed a statistically significant improvement.

The programme was designed to create an accessible internal resource that could recognise distress, offer initial support and facilitate contact with professional services. This rationale is consistent with preventive approaches in occupational health [18], but the present study did not measure individual exposure to promoter activities, number of contacts, referrals or intervention fidelity.

The MBI-GS distinguishes exhaustion, cynicism and professional efficacy [15,16]. Developmental models propose relationships among these dimensions, although their temporal ordering is not universal [19]. Previous work has also associated workplace aggression and value conflict with all three dimensions [20]. In the present data, cynicism and professional efficacy changed more clearly than exhaustion. One possible explanation is that baseline exhaustion was relatively low, but this interpretation is tentative and should not be treated as evidence of a reverse burnout sequence. Evidence linking exhaustion and workload with safety outcomes in healthcare also comes from a different occupational context [21].

The scores recorded for suicidal thoughts were low and showed no significant differences. No conclusions can be drawn regarding an improvement in suicidal thoughts. More generally, a three-year study on workplace promotion observed an increase in participation, but mixed results in terms of mental health, including differences by gender and by intervention component [22]. This study supports longer-term follow-up, whilst cautioning against the assumption that participation necessarily leads to widespread improvements.

The main strength of this project is access to an organisational population that is rarely represented in intervention research and the attempt to sustain training and follow-up rather than deliver a one-off activity.

It is therefore not possible to conclude with certainty that the training programme caused the changes observed. Other factors may have contributed, such as the attention paid by the researchers, the interviews, organisational changes, increased awareness amongst management, or other activities associated with the project. In this regard, it is worth noting that, in recent decades, deaths from road traffic accidents have fallen dramatically, particularly in developed countries. Improvements to infrastructure, the vehicle fleet, high-impact advertising campaigns and tougher penalties for offences all contributed to this outcome collectively. However, we cannot assess the impact of each of these measures in isolation. Greater awareness of mental health care, training within institutions and the dispelling of certain myths, enabling people to speak freely about the issue, undoubtedly contribute to a better response in this area.

These results point to a trend whereby training in basic mental health promotion knowledge and skills, aimed at company staff so that they can act as agents, may, over time, lead to improvements in the health of other members of the organisation [22].

Our initial expectations regarding the outcomes of the training were far more ambitious, but we have had to scale them back and adopt a humble approach. It is true that the organisations that received the most attention and support have benefited the most. But it is also true that this is a long-term study (at least six years) and that we believe the ongoing training of health promoters, and the impact this has on their organisations, will yield more conclusive results.

Furthermore, it should be noted that health variables take time to manifest themselves and, therefore, we understand that any improvement is not instantaneous but requires a process [11]. The data obtained during the first year of follow-up reflect a trend that will need to be corroborated over time [22].

The strength of this research lies in the fact that it is a study involving a hard-to-reach sample, and the results from the first year of follow-up show a clear trend towards improved mental health. It is hoped that this trend will be confirmed in the coming years and that it will help us to recognise that this type of intervention, through training, can contribute to an understanding that suicide prevention is one of the ultimate outcomes of genuine mental health promotion in different settings.

The limitations of the study are, on the one hand, that the participants were not randomly assigned to one of the two conditions (the organisations were randomly assigned, and the fact of belonging to one centre or the other determined their allocation to the control group or the intervention group) and, on the other hand, as this involved following subjects over time, it entailed the phenomenon of experimental attrition, significantly reducing the number of participants who completed the questionnaires at all three measurement points, despite the dissemination and reminder measures that were implemented within the organisations.

A common feature of the company assessed is that they serve as a preparatory environment enabling workers to enter the labour market in other organisations. Consequently, it is common for employees to leave once they secure contracts with other companies. Furthermore, the maximum length of stay in these integration company is four years. For both these reasons, staff turnover occurs more frequently over time than in other companies. This was observed in all five organisations. Furthermore, although follow-up activities (posters, emails) were organised, participation declined across all five centres, and it was not possible to include participants who had not been involved from the outset. This is highlighted as one of the study’s limitations.

Other significant limitations include the following: only five organisations were allocated; individuals within each organisation cannot be assumed to be statistically independent; only complete cases were analysed; attrition was substantial; promoter exposure and fidelity were not measured systematically; and multiple exploratory tests were conducted. Six of the seven promoters were human-resources managers, which may also have influenced accessibility and employee responses. However, The five centres belong to the same organisation of social enterprises, with common policies. Furthermore, it was found that the two groups did not differ significantly in terms of sociodemographic variables at the start of the study. The aim was to observe possible changes in health variables within those organisations where staff who had received training in this area were working.

## 5. Conclusions

The findings should be interpreted with great caution. Most of the results show favourable descriptive trends in the intervention group, but statistically significant results are only observed in a limited number of comparisons. Emotional exhaustion and suicidal ideation, in particular, do not show statistically significant improvements. Therefore, claims that the programme was ‘effective’ or that it improved mental health across the organisation go beyond the evidence currently available. It is for this reason that these results are presented as preliminary or exploratory evidence of potentially favourable trends.

Taking the above into account, we can conclude that the training of MHP in the workplace, as well as the monitoring of these actions, fosters a positive trend towards improved mental health over time, which encourages us to continue with both the training and monitoring activities.

We must not forget that health initiatives in general, and those relating to mental health in particular, require time and continuity before their benefits can be seen. It is not a question of carrying out one-off initiatives, but of implementing a sustained programme. Although it may seem somewhat costly in terms of effort and financial resources, it can prove far more effective for organisations to run a mental health promotion programme than to deal with the potential consequences [22].

## Figures and Tables

**Table 1:** Training programme for mental health promoters.

PSYCHOEDUCATION Sessions 1 and 2: Physical, psychological and social health. These sessions focus on knowledge of elementary psychological problems, risks, predisposing, precipitating and maintaining factors (discussion of examples provided by the participants). INDIVIDUAL WELLBEING Session 3: Responsibility for one’s own health. Self-care strategies: healthy lifestyles, rest habits, sport, nutrition (simple exercises to be copied). Sessions 4 and 5: Individual psychological techniques (breathing, relaxation, breaks at work). MENTAL HEALTH PROMOTION Session 6: Recognition of precursor signs of psychopathology, such as changes in behaviour, emotional expression, eating habits, toilet habits, hygiene habits, etc. Sessions 7 and 8: Wellbeing through social relationships; support dispensing techniques; role-play exercises covering how to deal with a range of different situations, in which the person is willing to cooperate or refuses to cooperate; training in empathy, intuition, respectful treatment and assertiveness skills (cross-disciplinary training over several sessions). PSYCHOSOCIAL FACTORS IN THE WORKPLACE Session 9: Stress and health. This session examines how stress and burnout are not individual problems, but organisational ones; training includes a practical description of techniques for assessing such issues and proposing solutions to management and staff. Sessions 10 and 11: Psychosocial risks in the workplace. This session expands upon identification and intervention tactics to eliminate or reduce such risks; practical tips and exercises using the organisation’s own data are provided. Session 12: Community resources and services, including description of services, invitation to professionals and associations.

**Table 2:** Participants in the baseline assessment.

	Total Sample	Intervention Group	Control Group	*p*-Value (CI)
	N = 87	N = 55	N = 32	
**Gender** (%, N)	55.0% F (47)	52.7% F (29)	56.2% F (18)	0.822
	45.9% M (40)	47.2% M (26)	43.7% M (14)	
**Age** (mean, SD)	41.7 (4.27)	40.31 (4.55)	41.94 (7.72)	0.370 (−1.34; 0.49)
**Disability**				
Sensory	16.1% (14)	14.5% (8)	18.7% (6)	0.692 (−4.35; 4.28)
Physical	31% (27)	29.1% (16)	34.3% (11)	0.081 (−1.55; 3.84)
Intellectual	5.74% (5)	7.3% (4)	3.12% (1)	0.059 (−1.72; 2.91)
No disability (risk of exclusion)	47.1% (41)	49.1% (27)	43.75% (14)	0.068 (−3.43; 5.06)
Convivence				
(family living %, N)	44.8% Yes (39)	47.3% Yes (26)	40.6% Yes (13)	0.946 (−101; 0.093)
	55.1% No (48)	52.6% No (29)	59.3% No (19)	
Seniority (mean, SD)	3.7 (1.32)	3.5 (1.2)	3.8 (1.37)	0.326 (−3.11; 0.038)
General Health (mean, SD)	24.01 (5.63)	25.62 (5.22)	23.94 (4.76)	0.844 (−12.33; 16.51)
Burnout (mean, SD)				
EE	18.24 (5.30)	18.71 (7.57)	17.14 (4.87)	0.073 (−8.53; 7.62)
CY	9.17 (4.24)	8.21 (3.77)	9.46 (4.19)	0.772 (−3.68; 5.11)
PE	8.01 (4.09)	7.69 (3.31)	8.39 (4.02)	0.418 (−1.722; −0.876)
SI (mean, SD)	0.085 (1.37)	0.089 (1.62)	0.079 (1.56)	0.646 (−4.01; −0.115)

EE: Emotional Exhaustion; CY: Cynicism; PE: Professional Efficacy; SI: Suicide ideation. F = female, M = male; % = percentage within their group; N = number of subjects; SD = standard deviation; CI = confidence interval. Degrees of freedom = 2.

**Table 3:** Health variable scores, at baseline (T0), six months (T1) and twelve months (T2).

	Intervention Group	Control Group	*p*-Value (CI)
	N = 55	N = 32	
General Health (Mean, SD)			
T0	25.62 (5.22)	23.94 (4.76)	
T1	23.92 (5.89)	24.03 (8.81)	
T2	22.38 (8.01)	24.19 (7.93)	
	χ^2^(2, 55) = 7.82, *p* = 0.094	χ^2^(3.32) = 8.68, *p* = 0.171	
T1–T0	−0.17	0.09	0.843
T2–T0	−0.32	0.24	**0.001**
T2–T1	−0.15	0.16	0.076
**Emotional Exhaustion, EE** (Mean, SD)			
T0	18.71 (7.57)	17.14 (4.87)	
T1	18.15 (6.21)	18.03 (6.72)	
T2	17.31 (5.48)	18.49 (7.70)	
	χ^2^(2, 55) = 5.90, *p* = 0.111	χ^2^(2, 32) = 7.88, *p* = 0.093	
T1–T0	−1.77	0.9	0.166
T2–T0	−1.57	1.35	0.491
T2–T1	−0.83	0.47	0.284
Cynicism, CY (Mean, SD)			
T0	8.21 (3.77)	9.46 (4.19)	
T1	7.41 (4.02)	8.86 (3.55)	
T2	6.37 (4.82)	10.01 (5.94)	
	χ^2^(2, 55) = 6.83, *p* = 0.050	χ^2^(2, 32) = 6.71, *p* = 0.830	
T1–T0	−0.60	0.55	0.081
T2–T0	−1.80	0.54	0.047
T2–T1	−1.0	0.14	0.050
Professional Efficacy, PE (Mean, SD)			
T0	7.69 (3.31)	8.39 (4.02)	
T1	8.32 (4.38)	8.01 (3.22)	
T2	9.51 (3.79)	7.57 (4.71)	
	χ^2^(2, 55) = 5.72, *p* = 0.033	χ^2^(2, 32) = 7.54, *p* = 0.773	
T1–T0	−0.61	0.37	0.184
T2–T0	−1.72	0.79	0.036
T2–T1	−1.20	0.43	0.052
Suicide Ideation, SI (Mean, SD)			
T0	0.089 (1.62)	0.079 (1.56)	
T1	0.067 (3.47)	0.088 (3.71)	
T2	0.054 (4.03)	0.085 (4.23)	
	χ^2^(2, 55) = 7.77, *p* = 0.069	χ^2^(2, 32) = 6.41, *p* = 0.099	
T1–T0	−0.21	0.09	0.058
T2–T0	−0.33	0.06	0.066
T2–T1	−0.13	0.03	0.281

EE: Emotional Exhaustion; CY: Cynicism; PE: Professional Efficacy; SI: Suicide ideation. N = number of subjects; SD = standard deviation; CI = confidence interval; T0 = baseline measurement; T1 = measurement at 6 months; T2 = measurement at 12 months. Degrees of freedom = 2.

## Data Availability

The data on which the results of this study are based are available upon request to the corresponding author. The data are not publicly available because they are part of an ongoing longitudinal study, in which new centres are being included and measurements are taken every six months. Using the questionnaires provided in the manuscript, any researcher can replicate the data in this study. The authors report the precedents for sample collection, inclusion criteria, and their establishment prior to data analysis, and report all study measures and analyses.
